# From Altered States to Altered Trajectories and From Molecules to Meaning: A Network Meta Analysis Mapping Comparative Efficacy of Psychedelic Assisted Therapies in Modern Psychiatry

**DOI:** 10.1192/bjo.2026.11141

**Published:** 2026-06-30

**Authors:** Gaurav Uppal, Naima Gul, Asha Dhandapani, Sathyan Soundararajan, Khushboo Kansal

**Affiliations:** 1 Satyam hospital, Ludhiana, India; 2 Leeds Community Healthcare Trust, Leeds, United Kingdom; 3 BCUHB, Wrexham, United Kingdom; 4 Amaha Health, Delhi, India

## Abstract

**Aims:**

Recently the therapies that utilize psychedelics have emerged as promising treatments for mood disorders, trauma-related disorders, and substance use disorders. While there is a growing body of research comparing various interventions directly, the majority of available research has been conducted in an indirect manner; therefore, Network Meta-Analysis (NMA), a statistical method for comparing the efficacy of multiple interventions at once, is well-suited to evaluate the available evidence and create a comparison of relative efficacy among the various psychedelic assisted interventions. This study aims to compare the efficacy of psychedelic-assisted interventions and provide a comparative efficacy ranking of each treatment modality using a Network Meta Analytic approach.

**Methods:**

A contrast-based NMA was used to synthesize randomized and controlled trials of psychedelic-assisted interventions. Each treatment node was created based upon the type of drug being utilized (psilocybin, ketamine, MDMA) and the specific therapeutic context (psychotherapy) along with comparator treatments (escitalopram, active placebo, and waitlist). Means and standard deviations (SD) of continuous outcomes were obtained, standardized (as Hedges g) and incorporated into a fixed-effect inverse-variance weightedmodel. Trials with crossover designs contributed their data prior to crossover. Trials that reported binary or time-to-event outcomes were incorporated into the geometric structure of the network. Surface Under the Cumulative Ranking Curve (SUCRA) was used to estimate rankings of each treatment.

**Results:**

A total of six studies comprised the network and all but one study had psilocybin-assisted psychotherapy as the focus of the study. All studies demonstrated that psychedelic-assisted treatments were more effective than control groups (pharmacological, active placebo, and waitlist). Study-level estimates of treatment effect favoured psilocybin (range=−0.6 to −1.4) when compared to all other treatments. Psilocybin at high doses was found to be more effective than low dose psilocybin in reducing symptoms. A large and statistically significant pooled summary effect favouring psychedelic-assisted therapy was observed (−0.89, 95% CI: −1.07 to −0.72). Using SUCRA, psilocybin-assisted psychotherapy ranked highest among all treatments examined and both MDMA-assisted psychotherapy and ketamine-assisted therapy ranked higher than all control condition treatments.

**Conclusion:**

In this network meta-analysis we present encouraging quantitative evidence supporting the clinical efficacy of psychedelic-assisted therapies, particularly psilocybin-assisted psychotherapy. These findings support the potential therapeutic utility of these treatments and suggest the need for additional head-to-head trials to provide more precise comparative effectiveness estimates and develop future clinical practice guidelines.

